# Pre- and post-pandemic risk perceptions and worries

**DOI:** 10.3389/fpsyg.2024.1412252

**Published:** 2024-07-12

**Authors:** Katharina Wolff, Svein Larsen

**Affiliations:** Department of Psychosocial Sciences, University of Bergen, Bergen, Norway

**Keywords:** Tourist Worry Scale, COVID-19, risk perceptions, pre- and post monitoring, travel and tourism

## Abstract

**Introduction:**

The present study is a cross-sectional investigation of worry and risk perceptions regarding various hazards and destinations, measured before, during, and after the COVID-19 pandemic.

**Methods:**

Questionnaire data were collected from tourists in Norway during the summer seasons of 2012 (*N* = 2,669), 2019 (*N* = 1,666), and 2022 (*N* = 956), and from a representative sample of Norwegians in 2020 (*N* = 1,003).

**Results:**

The results show a general decline in the level of worry and risk perceptions post-pandemic compared to those pre-pandemic, with the exception of infectious diseases, whose perceived risk slightly increased in 2022.

**Conclusion:**

The results highlight the importance of employing cross-sectional or longitudinal data to investigate changes in risk perceptions over time. The findings also indicate that pessimistic predictions of a continued decline in tourism appear to be unwarranted.

## Introduction

The COVID-19 pandemic has severely impacted travel and tourism, especially during periods of lockdowns and travel restrictions (UNWTO, 2023). Since the start of the pandemic, research has focused on understanding how risk perceptions regarding COVID-19 may influence tourist behavior and travel choices. Reported findings show that increased COVID-19 risk perceptions negatively affect travel intentions (Yoo et al., 2022; Golets et al., 2023; Fuchs et al., 2024). Moreover, tourists may have become more cautious by avoiding destinations perceived as high risk for COVID-19 (e.g., Orîndaru et al., 2021; Jeczmyk et al., 2023), and overall, travel is perceived as riskier during the pandemic (Villacé-Molinero et al., 2021; Susanti et al., 2023). Research also shows changes in tourist preferences due to COVID-19 risk perceptions. Hence, interest in certain destinations increased during the pandemic, while interest in other destinations decreased. For example, interest in nature-based tourism increased while interest in city-based tourism decreased (Huang et al., 2021; Hardt and Glückstad, 2024). While these results are interesting, they are all based on retrospective reports of perceived risk obtained during and after the pandemic. Various cognitive biases can influence risk perceptions that are assessed retrospectively, i.e., risk perceptions may appear elevated only after the hazard has occurred (Wolff et al., 2019). However, comparisons of before and after measures of the same hazard often show no increase in risk perceptions. To our knowledge, no research comparing risk perceptions and worries obtained before the pandemic to those obtained during and after the pandemic is available. This study is unique in that it aims to address this gap.

## Materials and methods

Data were collected through surveys conducted among tourists visiting Norway during the summer seasons of 2012, 2019, and 2022, with participants hailing from over 70 different countries. Additionally, in 2020, when data collection among tourists was impossible due to the pandemic, we obtained data from a representative sample of the Norwegian population through an online survey (see Table 1 for sample demographics).

**Table 1:** Sample demographics.

	**2012 Tourist sample**	**2019 Tourist sample**	**2020 Representative Norwegian sample**	**2022 Tourist sample**
***N***	**2,669**	**1,666**	**1,003**	**956**
Mean age (SD)	38.96 (16.33)	41.57 (16.63)	47.92 (17.10)	40.88 (16.90)
Females	46.7%	44.0%	50.5%	41.0%
(no) organized travel group	(80.4%) 18.5%	(81.6%) 17.8 %		(89.0%) 10.8%
**Last night's accommodation:**				
Camping	12.9%_a_	10.4%_ab_		14.4%_b_
Pension/hostel	20.3%_ab_	7.0%_a_		8.1%_b_
Hotel	30.0%_a_	34.2%_a_		44.9%_a_
Cruise ship	17.6%_a_	20.2%_b_		5.7%_ab_
Home	16.9%_ab_	27.5%_a_		26.6%_b_
**Top 10 Nationalities**				
	Germany (15.4%)	Germany (21.9%)		Germany (15.6%)
	UK (14.6%)	USA (16.4%)		USA (15.4%)
	USA (12.1%)	UK (8.7%)		France (8.4%)
	France (6.9%)	The Netherlands (5.8%)		Norway (6.7%)
	The Netherlands (5.9%)	Spain (4.1%)		UK (6.5%)
	Norway (5.4%)	France (4.0%)		The Netherlands (6.2%)
	Spain (3.5%)	Australia (3.7%)		Italy (6.2%)
	Italy (3.5%)	Belgium (2.9%)		Spain (5.6%)
	Australia (3.1%)	Poland (2.8%)		Switzerland (2.9%)
	China (2.9%)	Italy (2.7%)		Belgium (2.4%)

Proportions of accommodations sharing the same subscript are significantly different from each other across years (*X*^2^ (8, *N* = 5,266) = 350.03, *p* < 0.05 Bonferroni correction).

Risk perceptions for various hazards were assessed in 2012 and 2022 using the following questions, followed by a list of hazards: *How risky do you consider the trip you are on now to be concerning*… *terrorism or actions of war/food poisoning/infections or infectious diseases (such as for example SARS or HIV)/traffic accidents/violence or other forms of criminality/suffering accidents/petty crime/All in all, how risky do you judge this trip to be?*

Risk perceptions for various destinations were measured in 2019 and 2022 using the following question: *If you were to visit the following destinations as a tourist, how risky would you judge them to be? Consider each destination and rate it in relation to the general risk of unwanted events*. followed by a list of these destinations: *Norway and the Nordic countries/Germany, Austria, or Switzerland/Visiting larger cities in the USA/Roundtrip to Israel/Roundtrip to Turkey/Visiting larger cities in Europe/Musical and shopping in London/Roundtrip to the USA/Cultural trips to Spain*. Hazards and destinations were rated on a 7-point Likert-type scale, with 1 indicating *not risky* and 7 indicating *very risky*.

Tourist worries were assessed in 2019 and 2022 using the Tourist Worry Scale in samples of tourists, and in 2020, this factor was assessed within a representative Norwegian sample (Larsen et al., 2009; Wolff and Larsen, 2013).

## Results

Regression analyses were conducted to assess the predictive power of age, female gender, organized group travel, and year of data collection on risk perceptions for various hazards and destinations. The results showed a significant decline in risk perceptions for all hazards from 2012 to 2022, except for the perceived risk of infectious diseases, which increased significantly (see Table 2). Additionally, risk perceptions for six out of nine tourist destinations decreased, while they remained unchanged for three destinations from 2019 to 2022 (see Table 3). A MANCOVA was used to assess whether scores on the Tourist Worry Scale varied depending on the year of data collection while controlling for differences in age and gender distribution across the samples. The results also showed that scores peaked in 2020 but were significantly lower in 2022 than before the pandemic in 2019 and during the pandemic in 2020 (see Table 4).

**Table 2:** Regression analyses predicting risk perceptions for various hazards.

	**B**	**SEB**	**β**	***p***	***R* ^2^**
Terrorism or actions of war					0.01^*^
Age	−0.00	0.00	−0.06	< 0.001	
Female gender	−0.02	0.03	−0.01	0.57	
Organized group	0.00	0.04	0.05	0.01	
2022	−0.03	0.02	−0.03	0.05	
Food poisoning					0.02^*^
Age	−0.01	0.00	−0.10	< 0.001	
Female gender	−0.01	0.04	−0.00	0.82	
Organized group	0.24	0.05	0.09	< 0.001	
2022	−0.05	0.02	−0.04	0.02	
Infectious diseases					0.03^*^
Age	−0.00	0.00	−0.07	< 0.001	
Female gender	0.01	0.04	0.00	0.87	
Organized group	0.22	0.05	0.08	< 0.001	
2022	0.17	0.02	0.14	< 0.001	
Traffic accidents					0.01^*^
Age	−0.01	0.00	−0.11	< 0.001	
Female gender	0.02	0.04	0.01	0.64	
Organized group	0.05	0.06	0.02	0.36	
2022	−0.07	0.02	−0.05	0.004	
Violence or other crime					0.02^*^
Age	−0.01	0.00	−0.12	< 0.001	
Female gender	0.02	0.03	0.01	0.47	
Organized group	0.06	0.05	0.02	0.19	
2022	−0.09	0.02	−0.08	< 0.001	
Suffering accidents					0.02^*^
Age	−0.01	0.00	−0.10	< 0.001	
Female gender	0.03	0.04	0.01	0.49	
Organized group	0.09	0.05	0.03	0.09	
2022	−0.11	0.02	−0.09	< 0.001	
Petty crime					0.02^*^
Age	−0.01	0.00	−0.12	< 0.001	
Female gender	−0.01	0.04	−0.01	0.71	
Organized group	0.06	0.05	0.02	0.21	
2022	−0.09	0.02	−0.07	< 0.001	
All in all					0.02^*^
Age	−0.01	0.00	−0.11	< 0.001	
Female gender	−0.02	0.03	−0.01	0.49	
Organized group	0.12	0.04	0.05	0.004	
2022	−0.04	0.02	−0.05	0.009	

^*^p < 0.001 (Bonferroni correction for multiple analyses).

**Table 3:** Regression analyses predicting risk perceptions for various destinations.

	**B**	**SEB**	**β**	***p***	***R* ^2^**
Norway and the Nordic countries					0.01^**^
Age	−0.00	0.00	−0.01	0.61	
Female gender	0.01	0.05	0.01	0.79	
Organized group	0.25	0.07	0.08	< 0.001	
2022	−0.15	0.05	−0.08	0.002	
Germany, Austria or Switzerland					0.01^**^
Age	0.00	0.00	0.02	0.49	
Female gender	0.06	0.06	0.03	0.31	
Organized group	0.18	0.08	0.05	0.03	
2022	−0.17	0.06	−0.08	0.002	
Cultural trips to Spain					0.01
Age	0.00	0.00	0.04	0.15	
Female gender	−0.07	0.07	−0.03	0.31	
Organized group	−0.01	0.10	−0.00	0.94	
2022	−0.17	0.07	−0.06	0.01	
Musical and shopping in London					0.01^***^
Age	0.01	0.00	0.08	< 0.001	
Female gender	−0.15	0.07	−0.05	0.03	
Organized group	0.07	0.10	0.02	0.47	
2022	−0.19	0.07	−0.07	0.005	
Visiting larger cities in Europe					0.01^*^
Age	0.01	0.00	0.08	0.002	
Female gender	−0.05	0.07	−0.02	0.50	
Organized group	0.08	0.10	0.02	0.44	
2022	−0.14	0.07	−0.05	0.04	
Roundtrips in the USA					0.01
Age	−0.00	0.00	−0.02	0.48	
Female gender	−0.11	0.08	−0.04	0.16	
Organized group	0.13	0.11	0.03	0.24	
2022	0.16	0.08	0.05	0.03	
Visiting larger cities in the USA					0.01
Age	0.00	0.00	−0.00	0.94	
Female gender	−0.11	0.08	−0.04	0.15	
Organized group	0.12	0.11	0.03	0.31	
2022	0.17	0.08	0.06	0.02	
Roundtrip in Turkey					0.04^***^
Age	0.01	0.00	0.07	0.005	
Female gender	−0.23	0.07	−0.08	0.001	
Organized group	−0.04	0.11	−0.01	0.75	
2022	−0.55	0.07	−0.19	< 0.001	
Roundtrip in Israel					0.02^***^
Age	−0.00	0.00	−0.01	0.68	
Female gender	−0.33	0.08	−0.11	< 0.001	
Organized group	0.12	0.11	0.03	0.29	
2022	−0.21	0.08	−0.07	0.006	

^*^p < 0.05, ^**^p < 0.01, ^***^p < 0.001 (Bonferroni correction for multiple analyses).

**Table 4:** MANCOVA comparing mean values on the tourist worry scale for various years, controlling for age and gender distribution differences.

**(A)**									
	**2019 Tourist sample**		**2020 Representative Norwegian sample**		**2022 Tourist sample**		**Univariate**		
	***M***	**SD**	***M***	**SD**	***M***	**SD**	*F*_(2, 3436)_	***p***	η^2^
Stay awake and worry	1.41_a_	0.717	1.59_ab_	0.973	1.35_b_	0.704	24.03	< 0.001	0.01
More exposed to crime and accidents	2.13_a_	1.054	2.15_b_	1.164	1.95_ab_	1.035	12.91	< 0.001	0.01
Documents may contain mistakes	1.94_a_	0.923	2.11_a_	1.138	1.73_a_	0.890	49.36	< 0.001	0.03
Acts of terror or war at destination	1.61_a_	0.832	1.88_a_	1.062	1.34_a_	0.641	96.80	< 0.001	0.05
Petty crime	2.21_a_	0.982	2.55_a_	1.145	1.86_a_	0.898	123.62	< 0.001	0.07
Something may go wrong	1.82_a_	0.910	1.90_a_	1.036	1.62_a_	0.811	32.12	< 0.001	0.02
Get lost or lose travel companions	1.70_a_	0.909	1.76_a_	1.031	1.41_a_	0.750	48.13	< 0.001	0.03
The culture is strange and scary	1.37_a_	0.699	1.43_a_	0.784	1.19_a_	0.523	35.21	< 0.001	0.02
Mean values sharing the same subscript are significantly different from each other at *p* < 0.05 (Bonferroni correction).									
**(B)**									
**MANCOVA multivariate test**									
	**Multivariate**								
	***F*** **(Wilks'** λ**)**		**df**			***p***		η^2^	
Intercept	247.38		8, 3,429			< 0.001		0.37	
Age (covariate)	18.19		8, 3,429			< 0.001		0.04	
Gender (covariate)	3.85		8, 3,429			< 0.001		0.01	
Year	24.29		16, 6,858			< 0.001		0.05	

## Discussion

The comparison of pre-and post-pandemic risk perceptions for various hazards, destinations, and worries shows decreased scores in 2022, the first year after travel restrictions were lifted in many parts of the world, including most of Europe. The only exception to this trend is the perceived risk of infectious diseases, which was rated higher in 2022 than in previous years. The data also show that worries peaked during the pandemic in 2020. However, these data were collected from a representative sample of the Norwegian population who were not traveling at the time of data collection. Previous research has found that scores on the Tourist Worry Scale tend to be elevated in non-traveling samples compared to traveling ones (Larsen et al., 2009; Wolff and Larsen, 2017). The authors attribute this to the impact bias, a cognitive bias where individuals tend to overestimate the intensity of future emotions (Wilson and Gilbert, 2003). Therefore, the elevated worry scores observed in 2020 should be considered in the context of this bias.

The results are in line with earlier findings (Larsen et al., 2011; Wolff and Larsen, 2014, 2016), showing that increased risk perceptions and worries following dramatic events like natural disasters or terrorism are short-lived and limited to the affected destination. Risk perceptions might decrease in special circumstances even after a crisis (Wolff and Larsen, 2014, 2017, 2021). The findings are also in line with those of Grillo et al. (2023), who found robust risk habituation in COVID-19 risk perceptions in a longitudinal investigation. These authors found that risk perceptions decreased over time, even though objective threat indicators showed increased risk. This highlights the importance of longitudinal and cross-sectional risk measures to document changes in perceived risk. Research in the tourism domain appears to be dominated by studies that rely on people's memories for pre-pandemic risk assessment (Wolff et al., 2019). However, memories are likely flawed by rosy retrospection (Mitchell et al., 1997), which refers to people's tendency to rate events retrospectively more positively than they did during their occurrence. For example, respondents rated the world as safer in the past, while continuous and prospective measures of perceived risk remain constant (Brun et al., 2011; Wolff and Larsen, 2014).

It is also interesting to note that the level of risk perceptions and worries is not only “back to normal” in 2022 but is, in fact, lower than pre-pandemic. Such a decrease could be explained by the relief experienced following the pandemic or by mechanisms like the gambler's fallacy (see Wolff and Larsen, 2014, 2017, 2021). However, the observed decrease could partly be explained by a selection bias. People who choose to travel in 2022, almost immediately after the lifting of travel restrictions, might be the ones who worry the least and have the lowest risk perceptions. However, the number of international tourists arriving in Norway in 2022 was only 16% down from 2019 (UNWTO, 2023), and the composition of the pre and post-pandemic samples is very similar (see Table 1).

The findings are also in line with those of Larsen et al. (2011), who claimed that tourists do not judge the risk of various hazards independently of each other. They observed that, while risk perceptions for certain forms of travel increased, they decreased for other types of holiday. In the present data, risk perceptions for infectious diseases are increased. However, all other hazards are rated as less risky.

Naturally, the present data have their limitations in that the respondents were not asked about COVID-19 specifically. It was also not possible to conclude that the observed decline in risk perceptions and worries was caused by the pandemic. However, the fact that measures were obtained before and after the onset of the pandemic circumvents another problem that flaws the data collected post-pandemic only, e.g., rosy retrospection.

Another limitation concerns the fact that the samples differ slightly in some characteristics. While the analysis did control for possible effects of age, gender, and travel mode, there are also some variations in the accommodations utilized. Most notably, cruise tourists are underrepresented in the post-pandemic sample, which is surprising as cruise arrivals to Bergen increased in 2022 compared to the pandemic and pre-pandemic years (kystdatahuset, 2024). This discrepancy is, therefore, most likely an artifact of the data collection.

Finally, one might argue that, even though data were collected in a city (i.e., Bergen) in Norway, the country itself could be considered a destination low in COVID-19 risk. Therefore, it is uncertain whether the results can be generalized to destinations high in COVID-19 risk.

## Conclusion

In summary, our findings show a decrease in risk perceptions after the COVID-19 pandemic compared to before the pandemic. The results are in line with previous research showing that increases in risk perceptions following dramatic events are short-lived and limited. Findings are also in line with human nature in that people adapt to hazards and show decreased risk perceptions even when the objective risk remains constant or even increases (e.g., Grillo et al., 2023). This allows for the optimistic (or pessimistic) supposition that, as with earlier crises, travel behavior will return to pre-pandemic levels rather quickly. Our findings also highlight the importance of not relying solely on after-measures. Future research should examine whether risk perceptions display similar patterns in destinations perceived as higher risk than Norway.

## Data availability statement

The raw data supporting the conclusions of this article will be made available by the authors, without undue reservation.

## Ethics statement

Ethical review and approval was not required for the study on human participants in accordance with the local legislation and institutional requirements. Written informed consent from the patients/participants or patients/participants' legal guardian/next of kin was not required to participate in this study in accordance with the national legislation and the institutional requirements.

## Author contributions

KW: Conceptualization, Data curation, Formal analysis, Investigation, Methodology, Writing – original draft, Writing – review & editing. SL: Conceptualization, Investigation, Writing – original draft, Writing – review & editing.
